# Investigation of the Surface Characteristics of GCr15 in Electrochemical Machining

**DOI:** 10.3390/mi15091062

**Published:** 2024-08-23

**Authors:** Xuesong Liu, Guokang Su, Qingming Fan, Yongjun Zhang, Hua Chen, Chuanyun Zhang

**Affiliations:** 1 School of Mechatronic Engineering, Xi’an Technological University, Xi’an 710021, China; Liuxuesong1981@sohu.com (X.L.); su_guokang@163.com (G.S.); fanqingming@xatu.edu.cn (Q.F.); chenhua126@163.com (H.C.); 2 Shaanxi Engineering Research Center of Digital Precision Electrochemical Machining, Xi’an 710021, China; 3 School of Electro-Mechanical Engineering, Guangdong University of Technology, Guangzhou 510006, China; yjzhang@gdut.edu.cn

**Keywords:** electrochemical machining (ECM), GCr15 bearing steel, oxide film, removal, current density

## Abstract

Bearing steel (GCr15) is widely used in key parts of mechanical transmission for its excellent mechanical properties. Electrochemical machining (ECM) is a potential method for machining GCr15, as the machining process is the electrochemical dissolution of GCr15 regardless of its high hardness (>50 HRC). In ECM, NaNO_3_ solution is a popular electrolyte, as it has the ability to help in the nonlinear dissolution of many metallic alloy materials, making it useful for precision machining. However, due to high carbon content of GCr15, the electrochemical dissolution of GCr15 is unique, and there is always a black layer with high roughness on the machined surface, reducing the surface quality. In order to improve the electrochemical machining of GCr15 with a high surface quality, the surface characteristics of GCr15 in ECM were investigated. The anodic polarisation curve in the NaNO_3_ electrolyte was measured and electrochemical dissolution experiments were conducted with different current densities. SEM, XRD, and XPS were employed to analyse the surface morphology and composition formed on the machined surface at different current densities. The initial results showed that there were two parts (black part and bright part) formed on the machined surface when a short circuit occurred, and the test results suggested that the black part contained a mass of Fe_3_O_4_ while the bright part was composed of mainly Fe and Fe_3_C. Further investigation uncovered that a black flocculent layer (Fe_3_O_4_) always formed in a low current density (32 A/cm^2^) with high roughness. With the current density increased, the amount of black flocculent layer was reduced, and Fe_3_C particles appeared on the machined surface. When the current density reached 81 A/cm^2^, the entire flocculent oxide layer was removed, only some spherical Fe_3_C particles were inserted on the machined surface, and the roughness was reduced from Ra7.743 μm to Ra1.783 μm. In addition, due to exposed Fe_3_C particles on the machined surface, the corrosion resistance of the machined surface was significantly improved. Finally, circular arc grooves of high quality were well manufactured with current density of 81 A/cm^2^ in NaNO_3_ electrolyte.

## 1. Introduction

GCr15 bearing steel is widely used in the manufacturing of bearings in the automotive and aeronautic fields because of its excellent wear resistance, uniform hardness, and high elastic limit [1,2]. Compared with traditional mechanical methods, ECM exhibits robust processing prowess in certain characteristic applications. Wu Ming et al. employed mask electrolyte jet machining for the creation of micrometre-sized triangular and quadrilateral features on numerous metals [3]. Subsequently, to augment ECM’s manufacturing performance, machine learning was applied to forecast the contour of ECM [4]. Electrochemical machining (ECM) is the process of anodic dissolution with a specially formed cathode at large current densities and a strong electrolyte flow. It has the advantages of no tool wear and smooth surfaces, regardless of workpiece material hardness, and a relatively high machining rate for the machining of difficult-to-cut materials [5,6], including GCr15 (>50 HRC).

The electrochemical solubility of metals is significant for the machining quality of ECM. Scholars have paid increasing attention to investigating the electrochemical dissolution characteristics of metallic materials to improve their machining quality. Even with high electrolyte velocity, a black layer remained on the machined surface, leading to non-uniform dissolution [7]. Wang et al. investigated the electrochemical dissolution behaviour of Inconel 718 in NaNO_3_ solution [8] and found that Inconel 718 suffered serious selective corrosion due to the formation of a porous film in NaNO_3_ at a low current density (<5 A/cm^2^). For the ECM of iron-based materials, Song et al. and Fan et al. found that a black oxide layer was always formed [9,10] and the colour gradually darkened as current density increased from 2 A/cm^2^ to 30 A/cm^2^. To determine the composition of the black layer, Lohrengel et al. further studied the corrosion process of iron-based materials [11,12], and found that the black was mainly composed of Fe_2_O_3_ and Fe_3_O_4_ [13]. The black oxide layer on the machined surface would reduce surface quality and even the stability of the machining process [14]. Che et al. proposed electrochemical abrasive jet machining to remove the oxide film [15]. The flowing abrasives produced a cutting process role on the machining surface, and the oxide film could be effectively removed. Zhao et al. analysed the electrochemical dissolution features of horizontal and vertical sections of AM SUS 304 components at low current densities, discovering that the melt pool boundary is susceptible to dissolution and that the post-dissolution depressions, basins, and expansive materials segregate [16].

Numerous researchers have researched the electrochemical dissolution properties of easily passivated materials. Zhang et al. studied the electrochemical dissolution behaviour of tungsten during ECM [17]. They found that an oxide layer readily formed on the machining surface, avoiding the continuous dissolution in the NaNO_3_ solution. Liu et al. analysed the effect of anodic behaviour on ECM of TB6 titanium alloy in NaCl electrolyte [18]. He et al. examined the dissolution behavior of TA15 in NaCl solution under a low-frequency pulse current, revealing that TA15 would form a new passivation film during a longer pulse off time, accumulating the passivation effect, resulting in bumps and pits on the processed surface. This suggests that utilising long pulse conduction time can yield superior surfaces with distinct crystal structures [19]. Liu et al. scrutinised the electrochemical dissolution behavior of TB6 titanium alloy, exploring the surface dissolution processes at varying current densities. They discovered that, at high current densities exceeding 200 A/cm^2^, electrochemical dissolution displays enhanced uniformity, greatly reducing stray corrosion and achieving a minimal surface roughness of Ra 0.373 μm [20].

Although the electrochemical dissolution behaviours of numerous hard-to-machine materials have been reported, the electrochemical dissolution behaviour of GCr15 is seldom reported. As high-carbon steel, there are many cementites in the matrix, allowing for unique electrochemical dissolution behaviour. This paper mainly focused on investigating the electrochemical dissolution behaviour of GCr15 in NaNO_3_ solution, especially by analysing the surface characteristics under different current densities, which had a significant effect on the machining surface quality. The experiments were performed using various pulse current densities. Scanning electron microscopy (SEM), energy-dispersive X-ray diffraction (XRD), and x-ray photoelectron spectroscopy (XPS) were employed to analyse the surface morphology and the components formed on the machined surface. Following the investigation, the change in surface morphology and composition on the machining surface were well clarified and a method for the removal of the black layer was discovered. Finally, electrochemical impedance spectroscopy (EIS) was used to analyse the corrosion resistance of the machined surface.

## 2. Materials and Methods

### 2.1. Materials

The workpiece material is bearing steel (GCr15) made using a conventional forging method; the chemical composition of the material is listed in Table 1.

### 2.2. Methods

#### 2.2.1. Procedure for Testing Polarization Curve

An electrochemical workstation (Zennium E, Zahner, Kronach, Germany) was used to investigate the electrochemical dissolution characteristics of GCr15 in NaNO_3_ solution through detecting polarisation curves and open circuit potential (OCP). The three-electrode system was used in this experiment. The specimen was prepared using a piece size of 5 × 5 × 2 mm with a polished surface. A 15 × 15 mm platinum piece was employed as the auxiliary electrode, and, for the reference electrode, a saturated calomel electrode was adopted. The test parameters are listed in Table 2.

#### 2.2.2. Experiment

**Figure 1:**
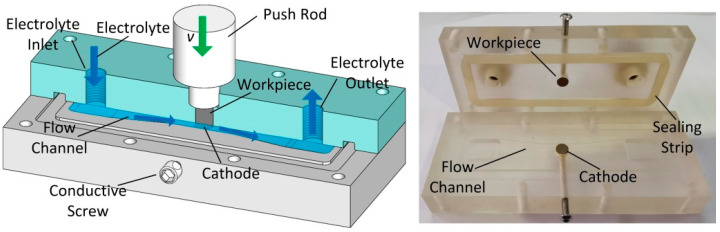
Schematic of the experimental set-up.

Figure 1 shows the schematic of set-up for obtaining a machined surface in ECM. Electrolytes with high velocities flowed into the machining gap along the flow channel, and the workpiece surface was dissolved when the tool and workpiece were connected with the cathode and anode, respectively. The rod-like cathode (copper) and workpiece were prepared with a diameter of 5 mm. The machining parameters for the ECM of the GCr15 are listed in Table 3.

The surface morphology was observed through a focused ion beam emission scanning electron microscope (LYRA3XMU, Tescan, Brno, Czech Republic). The XRD (D8ADVANCE, Bruker, Kalka, Germany) and XPS (Escalab 250Xi, Thermo Fisher, Waltham, MA, USA) were used to examine the corroded specimen surfaces.

## 3. Results and Discussion

### 3.1. Polarisation Curve

**Figure 2:**
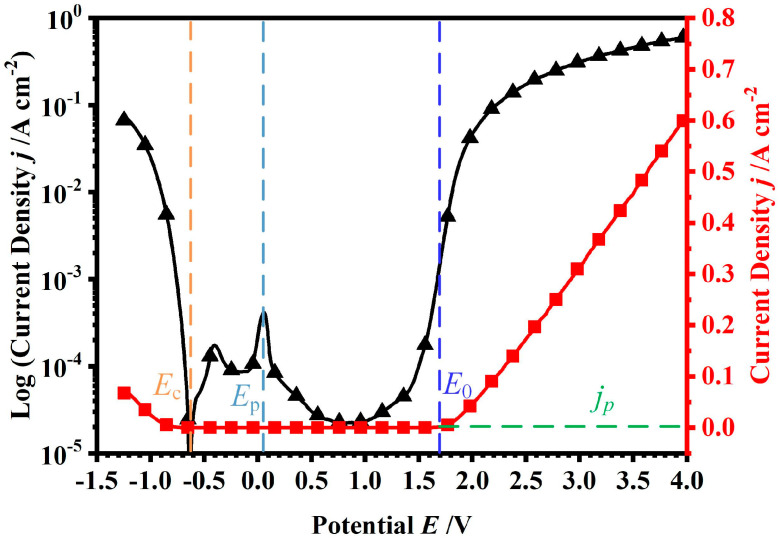
GCr15 bearing steel polarisation curves measured in 1.5 mol/L of NaNO_3_ solution.

To obtain the electrochemical characteristics of GCr15 in ECM, a polarisation curve of GCr15 in a solution of 1.5 mol/L of NaNO_3_ was recorded, as depicted in Figure 2. It showed that the polarisation curve included active dissolution, passivation, and trans-passive processes. The active dissolution potential was −0.62 V (*E*_c_), where the material began to react in the solution. When the potential increased, the current density decreased, and there was a peak in the current density at the potential of 0.05 V (*E*_p_). With the potential further increased, there was a sharp decrease in the current density, meaning that the passivation zone appeared, the oxide layer was formed on the workpiece, and the dissolution of material was hindered. When the potential reached 1.7 V (*E*_o_), the transpassive zone appeared, the passive oxide film was broken, and the material began dissolving. In general, the applied voltage is always higher than *E*_o_ for electrochemical dissolution. In this zone, because bearing steel is iron-based, the main reactions at the workpiece surface can be summarised as follows [21]:

(1) $${Fe}^{0}\to {Fe}^{2+}+2e^{-}$$

(2) $${Fe}^{0}\to {Fe}^{3+}+3e^{-}$$

(3) $$H_{2}O\to \frac{1}{2}O_{2}+2H^{+}+2e^{-}$$

### 3.2. Black Layer on the Machined Surface

#### 3.2.1. SEM

**Figure 3:**
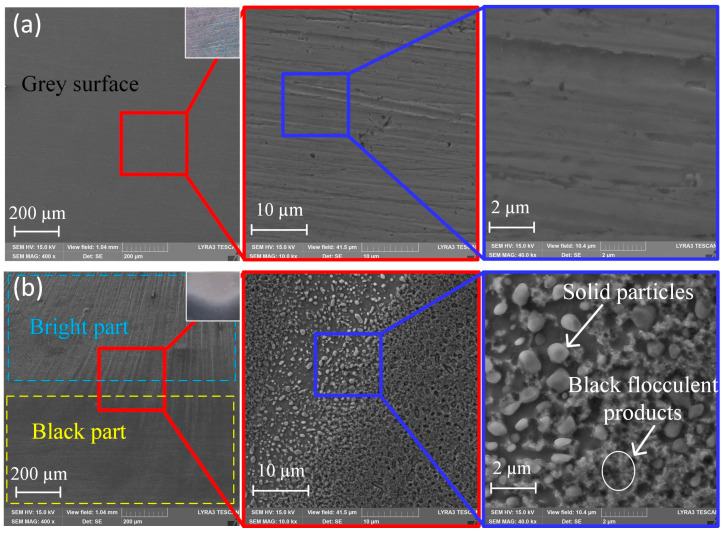
SEM of electrochemical machining GCr15: (**a**) unmachined surface; (**b**) machined surface with a short circuit.

**Figure 4:**
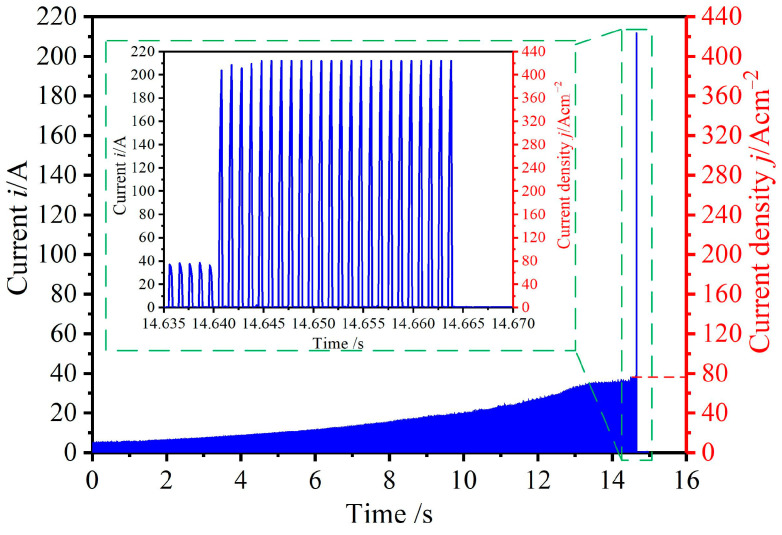
The current and current density of electrochemical machining GCr15.

In the initial experiment regarding ECM and GCr15, a voltage of 10 V was employed; other experiment parameters were as shown in Table 3. Figure 3a shows the SEM of the unmachined surface in which the entire surface was grey. However, when the feed rate was 1.8 mm/min, a short circuit occurred; the real-time current was recorded as in Figure 4, and the current was sharply increased. Then, there were two regions with different current densities (low and high current densities). An interesting phenomenon was found from the short-circuit machined workpiece (Figure 3b), in that the morphology of the machined surface was divided into two parts: a bright part and a black part. Moreover, the microstructures showed that there was a black flocculent layer on the black part, whereas many solid particles, not unlike pebbles, were evenly embedded in the bright part.

#### 3.2.2. XRD and XPS

**Figure 5:**
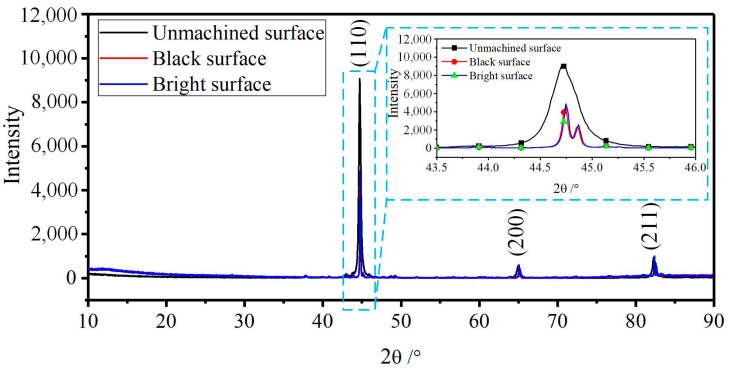
XRD patterns of GCr15 surfaces before and after ECM.

Figure 5 shows the XRD pattern of GCr15′s initial surface and its ECM surface (including black and bright parts). It can be seen that the diffraction peak was mainly formed by α-Fe, indicating that GCr15 was almost composed of polycrystalline ferrite and that the main crystal structures were unchanged after ECM. The enlarged image shows that the diffractions’ peak intensities on the machined surface (black and bright) were lower than that of the unmachined surface. In addition, another peak accompanied the diffraction peak, indicating that a new component appeared after machining.

**Figure 6:**
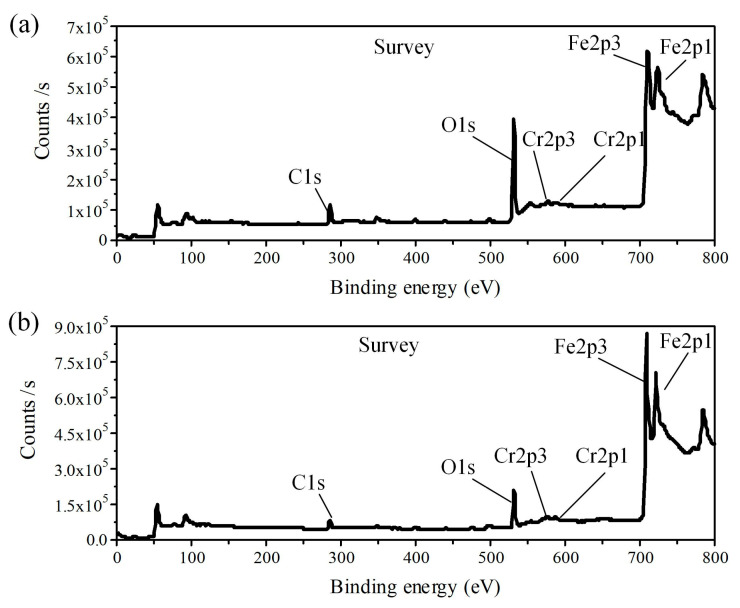
XPS spectrum of ECM GCr15 surfaces: (**a**) black surface; (**b**) bright surface.

**Figure 7:**
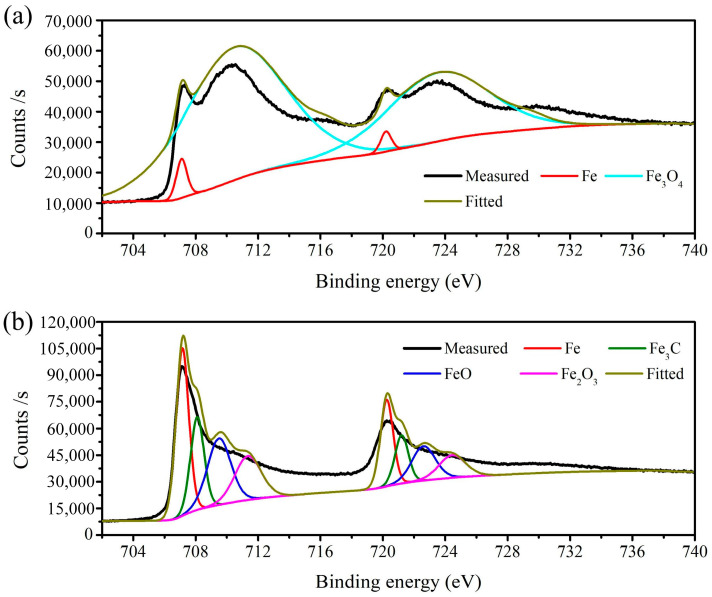
Fe 2p XPS spectrum of machined surface: (**a**) black surface; (**b**) bright surface.

XPS was employed to confirm the main composition of the component formed on the machined surfaces. Figure 6 shows the XPS spectrum of the black and bright machined surfaces of GCr15 after ECM. The main elements on the machined surface were Fe, O, and C, and the C and O content on the black surface was greater than that on the bright surface. Figure 7 shows the Fe 2p XPS spectrum of the black and bright surfaces machined by ECM. As can be seen in Figure 7a, the binding energy of 710.4 eV and 723.5 eV (cyan lines) correspond to Fe2p 3/2 and Fe2p 1/2, respectively, indicating that Fe_3_O_4_ was formed [22]. In addition, the peaks at 707.1 eV and 720.2 eV (red lines) represent the Fe matrix [23]. The results indicate that the black flocculent layer formed in the black region was mainly constituted by Fe_3_O_4_. As is known, the colour of Fe_3_O_4_ is black, displaying a black machined surface [24].

Figure 7b (i.e., bright surface) shows that the binding energy of 708.1 eV and 721.2 eV (olive lines) correspond to Fe2p 3/2 and Fe2p 1/2, respectively, indicating the existence of Fe_3_C on the bright surface [25]. In addition, the peaks at 709.4 eV and 722.5 eV (blue lines), which are consistent with the results of Mcintyre et al. [26], correspond to FeO on the bright surface. Finally, the satellite peaks at 711.4 eV and 724.8 eV (magenta lines) are characteristic of Fe_2_O_3_ [27], indicating the formation of Fe_2_O_3_ on the bright surface. According to the results of the Fe 2p XPS spectrum on the bright surface, Fe and Fe_3_C were the most frequently featured particles. Simultaneously, the spectrum contained a small amount of FeO and Fe_2_O_3_. Thus, the uniformly distributed spherical solid particles, embedded in the iron-based material, were Fe_3_C particles, as seen in Figure 3.

### 3.3. Influence of Current Density on the Black Layer

In the experimental results presented in Section 3.2, two different zones with different surface morphology and components appeared when a short circuit occurred. As can be seen in Figure 4, there was a high current density in the short circuit zone, which may have been the reason for the appearance of the two zones. To further investigate the surface characteristics on a machined surface, an experiment was designed to study the influence of current densities in those circumstances. In this experiment, pulse voltages of 10 V, 14 V, 18 V, and 22 V and a feed rate of 1.5 mm/min were employed; other machining parameters are presented in Table 3.

**Figure 8:**
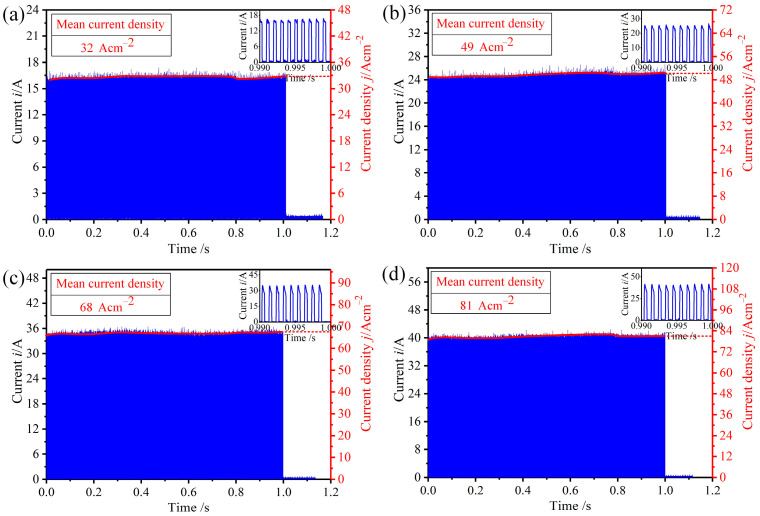
Current and mean current densities with different voltages: (**a**) 10 V; (**b**) 14 V; (**c**) 18 V; (**d**) 22 V.

**Figure 9:**
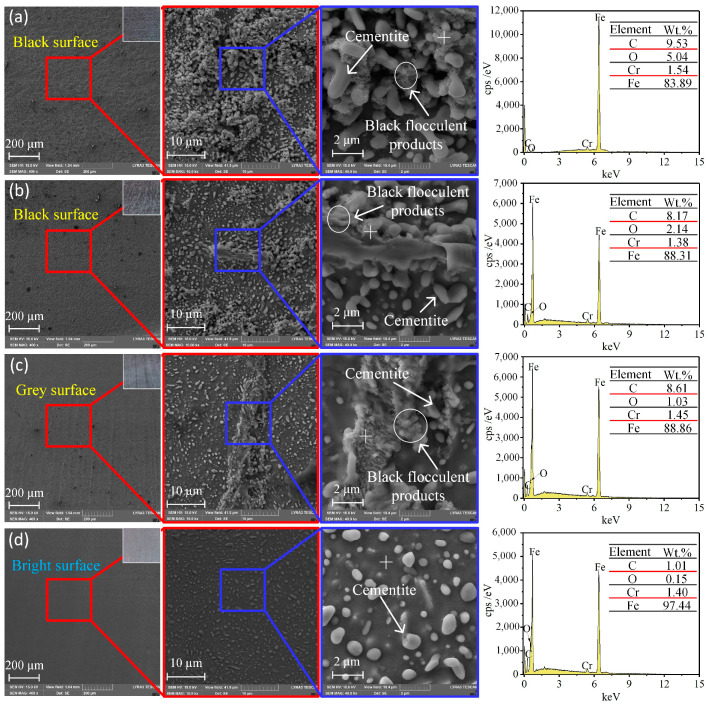
SEM and EDS of ECM GCr15 under different current densities: (**a**) 32 A/cm^2^; (**b**) 49 A/cm^2^; (**c**) 68 A/cm^2^; (**d**) 81 A/cm^2^.

**Figure 10:**
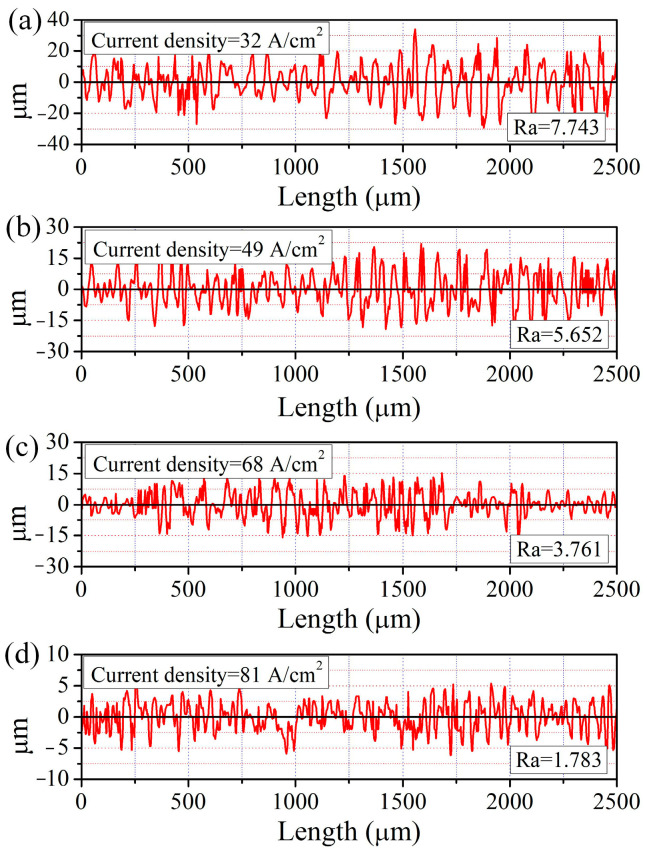
Roughness of ECM GCr15 under different current densities: (**a**) 32 A/cm^2^; (**b**) 49 A/cm^2^; (**c**) 68 A/cm^2^; (**d**) 81 A/cm^2^.

In the ECM process, the current before the end of machining directly affects the surface quality of the workpiece. The pulse currents at the last 1 s of machining were recorded, as in Figure 8, for which the current densities were calculated as 32 A/cm^2^ (10 V), 49 A/cm^2^ (14 V), 68 A/cm^2^ (18 V), and 81 A/cm^2^ (22 V), respectively. SEM and EDS images of the surface with different current densities are shown in Figure 9. With a current density of 32 A/cm^2^ (Figure 9a), the machined surface was black and covered with a flocculent layer (i.e., Fe_3_O_4_) in which the mass fraction of the O element was 5.04%. In addition, spherical solid particles (i.e., Fe_3_C) surrounded by the flocculent layer were found. As may be seen in Figure 10a, the roughness of the surface under this current density was Ra7.743. With the current density increased to 68 A/cm^2^, both the flocculent layer and the spherical solid particles were reduced. Simultaneously, the colour of the machined surface changed from black to greyish, as may be seen in Figure 9b,c. This can be explained by the mass fraction of the O element being reduced from 2.14% to 1.03%. Additionally, as indicated in Figure 10b,c, the roughness of the surface was reduced from Ra5.652 to Ra3.761. When the current density was increased to 81 A/cm^2^, the flocculent layer was fully removed, only some spherical solid particles were inserted, and the colour became bright, as shown in Figure 9d. This can be also verified by the mass fraction of O element being 0.15%. Additionally, as Figure 10d indicates, the roughness of the machined surface under a current density of 81 A/cm^2^ was low (Ra1.783).

On the basis of the experimental and measurement results, the surface morphology and composition change in GCr15 in NaNO_3_ solution can be explained as follows.

**Figure 11:**
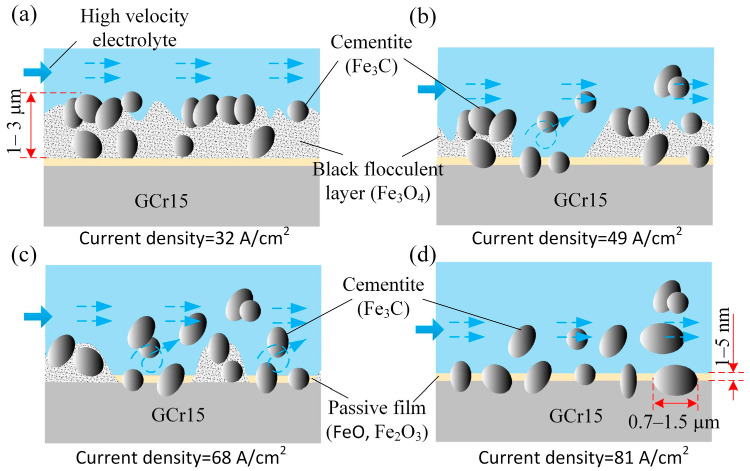
Tentative scheme of the sample surface during electrochemical machining: (**a**) 32 A/cm^2^; (**b**) 49 A/cm^2^; (**c**) 68 A/cm^2^; (**d**) 81 A/cm^2^.

At a current density of 32 A/cm^2^, a dense black flocculent layer and solid particles like pebbles adhered to the machined surface; the qualitative model is shown in Figure 11a. As indicated in Figure 6a and Figure 7a, the black flocculent layer was Fe_3_O_4_ and the solid particles were cementite (Fe_3_C). Because the size of the cementite was 0.7 μm to 1.5 μm (Figure 9a), the thickness of the black flocculent layer was 1 μm to 3 μm [28]. As it is difficult to flush away, a black surface with a high roughness remains.

As shown in Figure 9b,c, with the current density increased from 32 A/cm^2^ to 68 A/cm^2^, the amount of Fe_3_O_4_ and Fe_3_C on the machined surface was reduced, with the Fe_3_O_4_ disappearing completely in some areas (Figure 11b,c), leaving a surface quality with a low roughness.

From Figure 9d, it can be observed that, with the current density increased to 81 A/cm^2^, the dense black flocculent layer was completely removed. Instead, a thin passive film, including FeO and Fe_2_O_3_ (Figure 6b and Figure 7b), was formed, and some cementite was embedded in the GCr15. Thus, the surface was changed from black to bright and the machining quality was improved, with low roughness.

### 3.4. Electrochemical Impedance Spectroscopy

EIS is often used as a complementary method to test the corrosion characteristics of samples. This technique has been successfully applied to analyse the effects of anodic oxide films on corrosion resistance [29,30]. As machined surfaces are used directly in machinery, their corrosion resistance is an important index for the evaluation of the surface performance. Before each test, the sample was immersed in NaNO_3_ solution for 1 h to obtain a more stable OCP. The EIS measurements were performed at an OCP with an AC disturbance of 10 mV and a frequency of 10^−2^ Hz to 10^5^ Hz.

**Figure 12:**
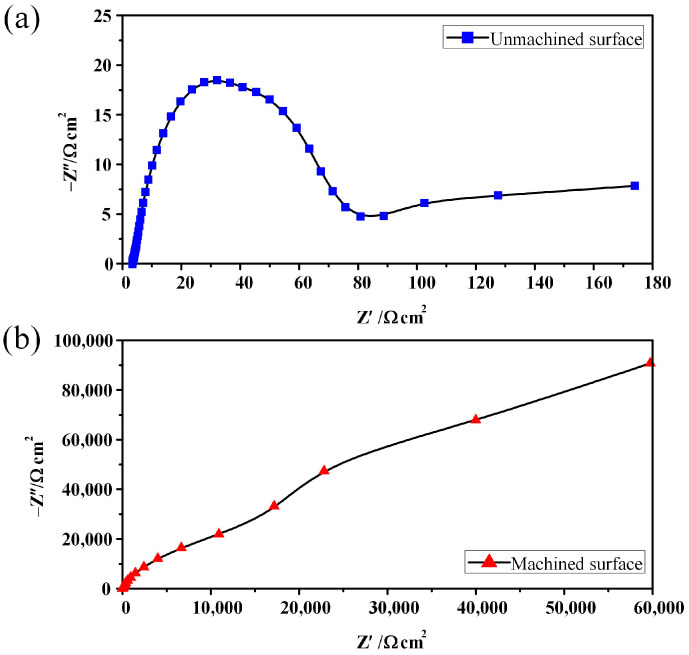
Nyquist plots for the surface of GCr15: (**a**) unmachined; (**b**) machined.

**Figure 13:**
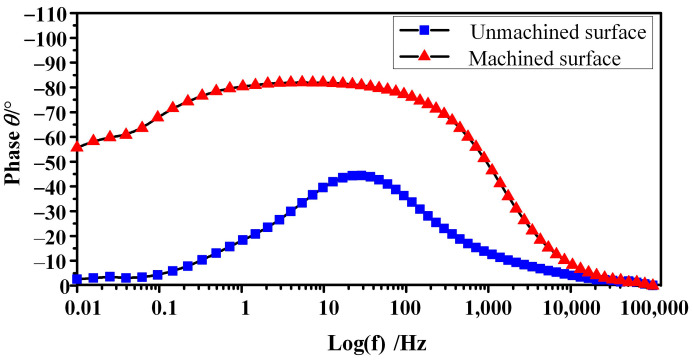
Bode representation of the phase angle as a function of frequency.

**Figure 14:**
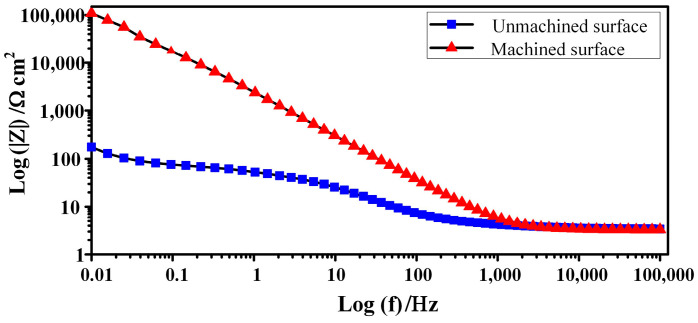
Bode representation of the impedance modulus as a function of frequency.

Figure 12, Figure 13 and Figure 14 show EIS plots for the unmachined and machined surface recorded in 1.5 mol/L NaNO_3_ solution. Generally, all the surfaces exhibited a capacitive response. The Nyquist plots for the surfaces are presented in Figure 12. As indicated in Figure 12a, reaction control and diffusion control featured in the high-frequency and low-frequency regions, respectively, whereas only diffusion control was found in all frequency regions (Figure 12b). The Nyquist radius of the machined surface was much larger than that of the unmachined surface, indicating that the polarisation resistance of the machined surface was much larger. Impedance mode in the low-frequency band is of great reference significance in the evaluation of corrosion resistance, and an impedance mode $\left|Z\right|$ at a frequency of 0.01 Hz was used to characterise it. As the Bode plots in Figure 12 and Figure 14 reveal, the peak phase angles and impedance moduli (log$\left(\left|Z\right|\right)$) of the machined surface were much larger than those of the unmachined surface. These results indicate that corrosion resistance was significantly improved on the machined surface of GCr15 by ECM, and this can be attributed to the exposed cementite on the surface [31].

### 3.5. Fabrication of Double Circular Arc Groove

**Figure 15:**
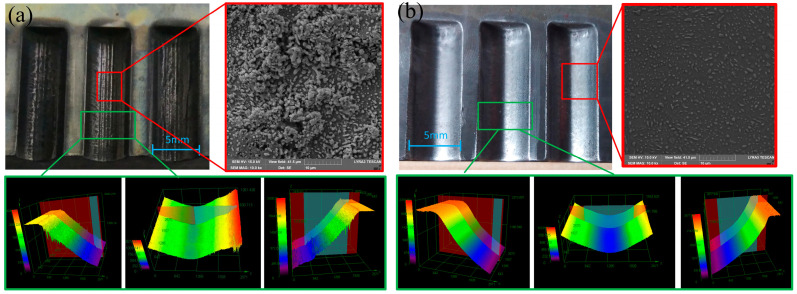
Images of circular arc grooves by electrochemical machining: (**a**) 10 V (32 A/cm^2^); (**b**) 22 V (81 A/cm^2^).

As shown in Figure 15, circular arc grooves were fabricated by electrochemical machining with specific parameters (i.e., a pulse frequency of 1 kHz, pulse duty cycle of 40%, electrolyte pressure of 0.5 MPa, and feed speed of 1.5 mm/min). In Figure 15a we can observe the results of an applied pulse voltage of 10 V; the machined surface was black and there were many spherical solid particles (i.e., Fe_3_C) surrounded by the flocculent layer on the machined surface, resulting in the rough surface. When the pulse voltage was 22 V, there was a blight machined surface and no flocculent layer, as shown in Figure 15b.

## 4. Conclusions

This study investigated the surface characteristics of GCr15 in electrochemical machining. The morphology and composition of the black layer was examined using SEM, XRD, and XPS. Furthermore, the influence of different current densities on the surface characteristics was studied. Based on our results and discussion, the conclusions can be summarised as follows:

1. The electrochemical dissolution characteristics of GCr15 show obvious passive and transpassive zones in NaNO_3_ solution, indicating that an oxide layer can be formed and removed during ECM.
2. In the case of a short circuit during ECM, black and bright parts on the machined surface were left. XRD and XPS results indicate that the black part was mainly covered by a dense black flocculent Fe_3_O_4_ layer. In contrast, many solid Fe_3_C particles were found in the bright part, though it also contained small amounts of FeO and Fe_2_O_3_.
3. In a current density of 32 A/cm^2^, a Fe_3_O_4_ layer was formed on the machined surface. With increasing current density, the thickness of the layer decreased. When the current density reached 82 A/cm^2^, the entire flocculent oxide layer was removed and only some spherical solid particles (Fe_3_C) were inserted, showing a bright surface with low roughness.
4. EIS results indicate that, due to the cementite exposed on the machined surface, its corrosion resistance was significantly improved over that of an unmachined surface.

## Figures and Tables

**Table 1:** Main chemical composition of GCr15.

Fe	C	Cr	Mn	Si	Others
Balance	1.01	1.46	0.36	0.25	<0.05

**Table 2:** Parameters for polarisation curve measurement.

Parameter	Value
Electrolyte (wt.%)	1.5 mol/L, NaNO_3_
Measuring potential (V)	−1.25~4
Scan rate (mV/s)	1
Temperature (°C)	25

**Table 3:** Experimental parameters for ECM of GCr15.

Parameter	Value
Electrolyte, NaNO_3_ (mol/L)	1.5
Applied voltage (V)	10, 14, 18, 22
Pulse duty cycle (%)	40
Pulse frequency (kHz)	1
Feed rate (mm/min)	1.5, 1.8
Initial gap (mm)	0.2
Electrolyte pressure (MPa)	0.5
Electrolyte temperature (°C)	25

## Data Availability

Data are contained within the article.
